# Incidence and Clinical Relevance of Incidental Papillary Carcinoma in Thyroidectomy for Multinodular Goiters

**DOI:** 10.3390/jcm12082770

**Published:** 2023-04-07

**Authors:** Aldo Bove, Roberto Manunzio, Gino Palone, Raffaella Marina Di Renzo, Giulia Valeria Calabrese, David Perpetuini, Mirko Barone, Stella Chiarini, Felice Mucilli

**Affiliations:** 1 Department of Medicine, Dentistry and Biotechnology, University “G. d’Annunzio” of Chieti-Pescara, 66100 Chieti, Italy; 2 Department of Neuroscience and Imaging, Institute for Advanced Biomedical Technologies, University G. D’Annunzio of Chieti-Pescara, Via Luigi Polacchi 13, 66100 Chieti, Italy; 3 Department of Medicine and Ageing Sciences, University “G. d’Annunzio” of Chieti-Pescara, 66100 Chieti, Italy

**Keywords:** total thyroidectomy, papillary cancer, microcarcinoma

## Abstract

Introduction: Patients undergoing a total thyroidectomy for multinodular goiter typically have a long clinical history of the disease. They often come to surgery for compression symptoms, with no suspicion of neoplastic disease. For these patients, the incidence of microcarcinomas is high, even though we know that this does not affect subsequent therapies and long-term survival. On the other hand, when a true incidental carcinoma is present, the patient requires specific therapy and long-term follow-up. The purpose of the study was to identify the incidence of incidental carcinomas in the high prevalence region of goiter, the clinical-pathological characteristics of the tumor, and the therapeutic implications. Method: This is a retrospective study, from January 2010 to December 2020, on a case series of 1435 total thyroidectomies for goiters. All patients had a preoperative diagnosis of a benign disease. Gender, mean age, and mean duration from the initial diagnosis of goiter were evaluated along with the number and frequency of fine needle aspirations carried out. On the basis of the histological examination, the incidence of incidental carcinoma was then assessed (diameter ≥ 10 mm) as well as the incidence of microcarcinoma (diameter < 10 mm), the pathological characteristics (multifocality, capsular invasion), and the subsequent prescribed therapies. Results: Patients with incidental carcinoma numbered 41 (2.8%%), 34 women and 7 men. The mean age was 53.5 years, while the patients diagnosed with microcarcinoma were 88 (6.1%). The mean duration of the disease from initial diagnosis was 7.8 years. On average, these patients underwent 1.8 fine needle aspirations during the course of the disease, almost exclusively in the first four years. The mean diameter of the tumor was 1.35 cm (±0.3). Multifocality was present in six patients, while only one patient presented capsular invasion. The chi-square test delivered a significant dependence on gender in terms of the incidental diagnosis after Yates correction (chi-stat = 5.064; *p* = 0.024), highlighting a higher incidence in the female population. All patients underwent subsequent metabolic radiotherapy. The mean follow-up was 6.3 years and in the 35 patients examined, none displayed any recurrence of the disease. Conclusions: Incidental carcinoma is not uncommon in patients who have undergone total thyroidectomy for goiters. It must be differentiated from microcarcinoma for its therapeutic implications and the follow-up of the patient. Statistical analysis has shown that the only significant variable is gender. In a goiter area, the careful monitoring of patients is required to highlight suspicious clinical–instrumental aspects that may appear even several years after the initial diagnosis.

## 1. Introduction

A multinodular goiter is frequent in iodine-deficiency areas and is often accompanied by a long clinical history [1]. It emerges during surgery for compressive symptoms on the trachea and esophagus, sometimes with alterations in the functionality of the thyroid.

Generally, total thyroidectomy is the correct surgical approach [2]. However, in recent years, a new approach called active surveillance has been suggested as an alternative to surgery. Active surveillance involves closely monitoring the tumor with regular check-ups, blood tests, and ultrasounds, but not immediately removing the tumor. If the tumor shows signs of growth or spreads to other parts of the body, then treatment may be recommended, such as surgery or radiation therapy [3]. This approach is based on the idea that some thyroid cancers, including papillary microcarcinoma, are so slow-growing that they may never cause harm to the patient, and that treatment may cause unnecessary risks and side effects. However, it is important to note that this approach requires careful monitoring and evaluation by a medical professional [4].

Much has been debated regarding the possible correlation between goiters and thyroid cancer. Published data are conflicting, with the incidence rate ranging between 3 and 35% [5]. This discrepancy also derives from a possible clinically insignificant difference between microcarcinomas and real thyroid tumors that require specific therapies and appropriate follow-up. Although the guidelines of the American Thyroid Association define microcarcinoma tumors as those of a size of less or equal than 1 cm in diameter, other parameters may affect this definition, including: capsule infiltration, multifocality, and the presence of lymph node metastases [6].

Fine-needle aspiration (FNA) remains the most accurate method for the diagnosis of thyroid cancer, with a minimal incidence rate (around 3%) of false negatives [7]. Unfortunately, facing multinodular goiters, FNA may not be used for diagnosis in almost 25% of cases due to the difficulty of reaching deep nodules or due to the contiguity of several nodules [8]. Furthermore, patients diagnosed with a multinodular goiter tend to decrease instrumental controls over time, often several years.

Protective effects have emerged in cases of hyperthyroidism due to thyroid stimulating hormone (TSH) suppression, which seems to delay the evolution of the neoplasm [9].

The aim of the study is to evaluate the incidence of incidental carcinoma in an area with a high goiter presence, the clinical–pathological characteristics of the tumor, and other relevant therapeutic implications.

## 2. Materials and Methods

This retrospective study was carried out from January 2010 to December 2020 in the surgical divison of the University “G. d’Annunzio” of Chieti-Pescara (Chieti, Italy), on 1435 thyroidectomy operations for goiters, out of a total of 1635 operated cases. Notably, 200 patients were not included in this retrospective study because their clinical exams and general data were not available.

All patients had a preoperative diagnosis of benign pathology, and all underwent total thyroidectomy, with prior written informed consent. A total of 1115 patients (77.7%) showed a euthyroid goiter, while 320 (22.3%) showed toxic thyroid disease and were treated with antithyroid drugs and operated under euthyroid conditions.

Variables such as gender, mean age, and disease length were assessed. The number and frequency of FNA performed was also taken into consideration.

Based on the final histological examination, the incidence of incidental carcinoma, defined as a tumor with a diameter bigger or equal than 10 mm; the incidence of microcarcinoma (diameter < 10 mm); the pathological characteristics of the tumor (multifocality and capsular invasion); and the subsequent therapies performed were counted.

Possible risk factors in patients with incidental cancer were evaluated.

### Statistical Analysis

To assess the influence of gender, age, duration of the disease, and number of FNA performed after the incidental diagnosis, a chi-square test of independence was carried out. The chi-square test is a nonparametric inferential statistical test that identifies the relationship between two categorical variables in a population. Specifically, if two variables are interdependent, the probability that one variable will have a particular value is dependent on the value of the other variable. Therefore, the test compares the observed frequencies with the frequencies that would be expected if the two variables were unrelated: when there is no relationship between the variables, the observed and expected frequencies will be comparable. In this study, the null hypothesis of the chi-square test demonstrates that the incidence of the disease is independent of gender in the examined population (95% confidence level, α < 0.05). In order to reduce the risk of incurring a type 1 error, the Yates correction was applied to the results.

Moreover, to investigate a possible effect of the age of the patients of the onset of the tumor in the male and female groups, a *t*-test for independent samples was performed. Notably, the normality of the age distribution of the two groups was investigated through the Shapiro–Wilk test. The null hypothesis of the Shapiro–Wilk test is that the distribution of the population is normal. In our study, the age distribution of the male and female groups resulted in a normal distribution (*p* > 0.05).

## 3. Results

**Figure 1:**
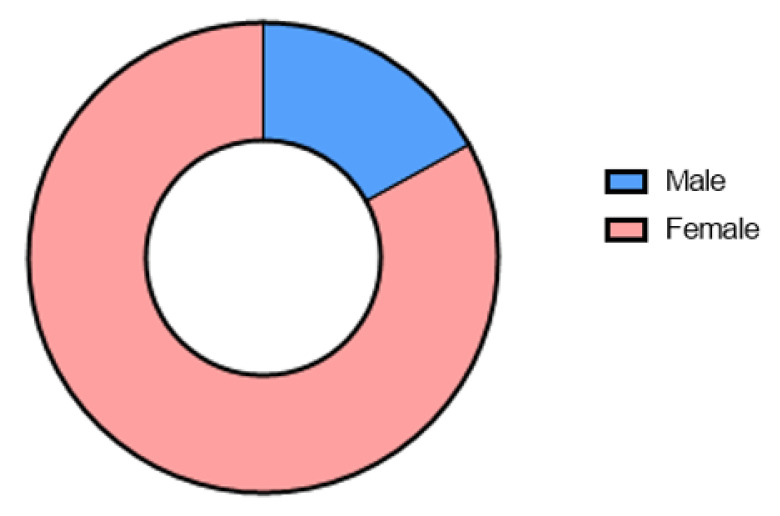
Tumor gender distribution. The figure shows the percentage of incidence of thyroid cancer in male (blue) and female (pink).

Out of 1435 patients, 1125 were female (78.4%) and 310 were male (21.6%), as reported in Figure 1.

**Figure 2:**
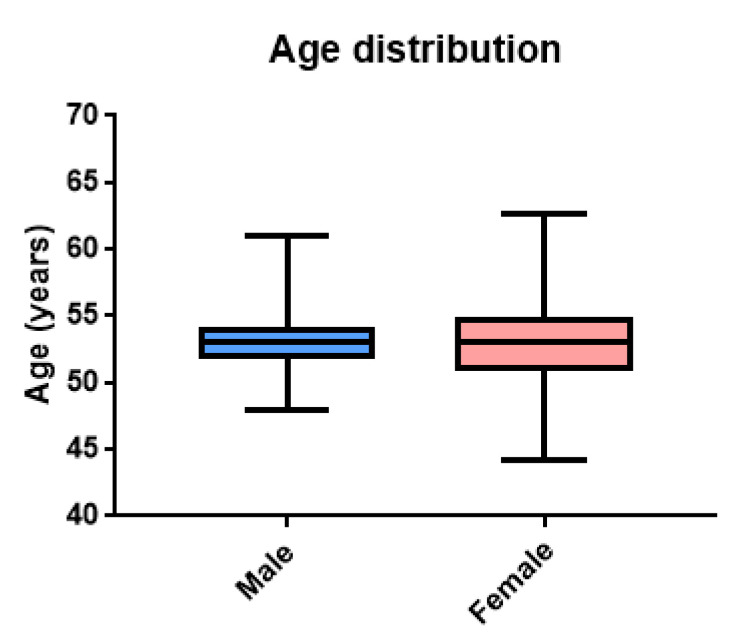
Age distribution of patients diagnosed with incidental cancer categorized by gender. The mean value of the age is not statistically different between the male and female groups (t = 0.661; *p* = 0.512).

Patients diagnosed with incidental cancer were 41 (2.8%), of which 34 were female and 7 were male. In these patients, the mean age was 53.5 (±2.8) for the male group and 52.95 for the female group, as reported in Figure 2. The mean age of the two groups was not significant (male vs. female, t = 0.661; *p* = 0.512).

We also found 88 patients (6.1%) diagnosed with microcarcinoma (diameter < 10 mm).

The mean duration of the disease from the initial diagnosis was 7.8 years (±3.1). On average, all patients underwent 1.8 (±1.2) FNA almost exclusively in the first four years after diagnosis. The mean tumor diameter was 1.35 cm (±0.3), comprising papillary carcinomas and one follicular tumor. Staging involved 32 patients in T1, 8 in T2, and 1 in T4. There were no significant differences in the percentage of incidental carcinomas between euthyroid patients (32 patients) and hyperthyroid patients (9 patients).

In the study sample, 15 male patients received an incidental diagnosis out of a total of 294, whereas among the 1158 female patients, 114 incidental diagnoses emerged (Table 1). The chi-square test delivered a significant dependence on the gender of the incidental diagnosis after Yates correction (chi-stat = 5.064; *p* = 0.024), highlighting a higher incidence in the female population, while age, the duration of the disease, and the number of needle aspirations did not turn out to be significant factors.

Multifocality was diagnosed in six patients (0.42%), whereas only one patient showed capsular invasion. All the patients subsequently underwent, on the indication of the radiologist, radio-metabolic treatment. In accordance with the guidelines of the AIOM (Associazione Italiana di Oncologia Medica), in patients at a medium-high risk of disease recurrence, after initial surgical treatment, we began with low doses of radioiodine (about 30 mCI), then progressed to high doses of radioiodine (≥100 mCI), depending on the patient’s histotype and metabolic response. The mean follow-up was 6.3 years (±2.7), and no recurrence was found in the 35 controlled patients.

Statistical analysis demonstrated that gender represents a risk factor for the development of incidental carcinoma, while age, the duration of the disease, and the number of needle aspirations did not turn out to be significant factors.

## 4. Discussion

The correlation between goiters and thyroid cancer remains an open question [10]. The incidence is certainly not minimal and largely depends on the possible presence of so-called microcarcinomas. The clinical relevance of these tumors is still controversial. In most cases, they are not included in the category of true carcinomas, as in guidelines of the American Thyroid Association [11]. However, some authors report case studies with incidental microcarcinoma with local invasiveness aspects that would indicate an aggressive tumor [12].

We aimed to study the incidence of incidental thyroid carcinoma in an area endemic for the presence of a multinodular goiter. Our sample represents a 10-year homogenized series in which all patients underwent total thyroidectomy for a preoperative benign disease. A total thyroidectomy was considered to be the gold standard for the treatment of a multinodular goiter.

Patients with thyroid goiters reported a long clinical history (in the considered study sample, this was around 8 years) and opted for surgery after compression syndromes or functional alterations [13]. The length of the disease is a factor that often leads patients to remain silent and attempt to live with it.

Importantly, the utility of FNAC in the diagnosis of carcinoma in multinodular goiter remains controversial. In fact, Lasithiokatis et al. reported a suspicious pattern in only 6.7% of cases [14], while Baier et al. reported a 12% false negative rate [15].

The difficulties in identifying possible dangerous nodules in the context of multinodular pathology are well known, both due to the contiguity of several nodules and due to the possible deep position of the nodule itself [16]. Notably, recent studies have investigated the possibility of employing liquid biopsy as a non-invasive procedure for the diagnosis and follow-up of thyroid cancer [17]. Specifically, liquid biopsy is a non-invasive diagnostic test that involves analyzing a sample of a patient’s blood or other body fluids to detect small amounts of tumor DNA or other biomarkers. It is a promising approach for cancer diagnosis and monitoring, and it has the potential to improve patient outcomes by enabling earlier detection and more personalized treatment. Unlike traditional biopsies, which involve surgically removing a tissue sample from the tumor, liquid biopsies are less invasive and do not require surgery [18].

In our experience, the incidence rate of microcarcinomas was 6.1% (88 patients), while true thyroid cancer was present in 2.8% (40 patients) of cases.

We believe it is important to make this subdivision because the therapeutic and follow-up problems are completely different [19].

Many reports fail to make this differentiation, leading to the possible misinterpretation of the analysis of the results [20]. The mean diameter was 1.35 cm, and the stage was T1 in 32 patients, T2 in 8 patients, and T4 in 1 patient (no patients in T3). This led, on the indication of the radiologist, to the need for radiometabolic treatment, which is obviously not suggested for microcarcinomas. Importantly, the thyroid cancers ranged from stages 1 through 4: the lower the number, the less the cancer has spread. The fact that the majority of the study sample consisted of T1 and T2 stages is attributable to the thorough screening of pathology in this iodine-deficient region. As a result, it is easier to evaluate individuals with a lower cancer stage due to the screening techniques. It is important to note that most participants were ignorant of the pathology prior to the histologic examination of the surgical sample. We compared certain parameters, such as sex, age, the number of needle aspirations carried out, and the duration of the disease of patients with incidental carcinoma, as possible risk factors.

Statistical analysis revealed that the only significant variable was gender.

Many authors report that age [21] and the presence of autoimmune disease [22] as risk factors, but supporting data are discordant and scarce. Segal et al. do not recognize Hashimoto’s thyroiditis as a condition favoring the onset of carcinoma [23], while Di Pasquale et al. believe that thyroiditis may be a precursor of carcinoma [24].

Thyroid disease is certainly prevalent in females, even if it does not seem to have a negative prognostic value with respect to the prognosis of thyroid cancer [25].

Both the duration of the pathology and the number of needle aspirations did not show statistical significance in the occurrence of incidental carcinoma.

A subsequent radiometabolic treatment was necessary in all 42 cases of incidental carcinomas, demonstrating the advanced stage of the disease regardless of the good, long-term prognosis.

Indeed, a distinction should be made between microcarcinomas and proper incidental carcinomas. Generally, for patients with multinodular goiters, the detection of incidental papillary thyroid cancer may require further evaluation and management, depending on the size and characteristics of the tumor. In addition, although age and other well-known risk factors are relevant, the female gender turned out to be the most significant variable. In the clinical practice, patients with multinodular goiter must undergo a stricter follow-up. In this perspective, it is important for healthcare providers to discuss the risks and benefits of different management options with patients and to involve them in the decision-making process. The limits of our study are the retrospective evaluation, the small number of cases compared to the total sample, and the lack of a control group, which made the statistical study less significant. In this regard, it should be highlighted that this study is exploratory in nature and aims to generate insights concerning a particular aspect of thyroid cancer, namely the incidence in this kind of population. Indeed, the absence of a control group limits the ability to draw causal inferences and make definitive conclusions about the association between papillary carcinoma and multinodular goiters. Another limit of the study is the infeasibility of implementing a predictive model of the presence of papillary carcinoma in the multinodular goiter population. In fact, the variables considered in this retrospective study are gender, mean age, and mean duration from the initial diagnosis of goiter; hence, they cannot be considered to be predictable variables of the pathology—only inferences regarding the incidence of the pathology related to these features could be investigated. However, the results suggest that incidental thyroid carcinoma is not an uncommon outcome for patients who have been operated on for multinodular goiters.

Microcarcinomas that do not require specific treatments and follow-ups are certainly to be distinguished. There is a high possibility of missing a pre-operative diagnosis, and it is therefore necessary to monitor these patients more accurately over time through needle aspiration in not only the dominant nodule but also those that appear subsequently.

The majority of patients diagnosed with incidental carcinoma after total thyroidectomy for goiter require radiometabolic therapy, demonstrating the clinical relevance of the disease. These patients must be monitored over time and only a long follow-up process can provide us with precise indications in terms of prognosis.

## 5. Conclusions

Incidental carcinoma is not uncommon in patients who have undergone total thyroidectomy for goiters. It must be differentiated from microcarcinoma, which has different therapeutic implications and follow-ups for the patient. Statistical analysis has shown that the only significant variable is gender. In a goiter area, the careful monitoring of patients is required to highlight suspicious clinical–instrumental aspects that may appear even several years after the initial diagnosis.

## Figures and Tables

**Table 1:** Incidental cancer by histological type and gender distribution.

	Number of Cases		Percentage
Incidental Carcinomas	41		2.8%
Microcarcinoma	88		6.1%
Total	129		8.9%
	Male	Female	
	15	114	

## Data Availability

The data are available on request to the corresponding author.
